# Ethanolic extract from *Sophora moorcroftiana* inhibit cell proliferation and alter the mechanical properties of human cervical cancer

**DOI:** 10.1186/s12906-024-04502-5

**Published:** 2024-06-03

**Authors:** Manli Guo, Dingcheng Guo, Lingzi Liao, Xiao Zhang, Zhilong Wang, Qiaozhen Zhou, Ping Chen, Ruiping Li, Bing Han, Guangjie Bao, Baoping Zhang

**Affiliations:** 1 Key Lab of Oral Diseases of Gansu Province, Northwest Minzu University, Northwest new village No.1, Lanzhou, 730030 PR China; 2 School (Hospital) of Stomatology, Lanzhou University, Donggang West Road 199, Lanzhou, 730000 PR China; 3 Gansu Province Key Lab of Maxillofacial Reconstruction and Intelligent Manufacturing, Donggang West Road 199, Lanzhou, 730000 PR China; 4 Chengdu Stomatological Hospital, NO. 17, South Section of Chunxi Road, Jinjiang District, Chengdu, 610020 PR China

**Keywords:** Cervical cancer, *Sophora Moorcroftiana*, Ethanol extract, Cell proliferation

## Abstract

**Background:**

Cervical cancer is one of the most common gynecological malignancies. Previous studies have shown that the ethanol extract of *Sophora moorcroftiana* seeds (EESMS) possesses an antiproliferative effect on several tumors in vitro. Therefore, in this study, we assessed the impact of EESMS on human cervical carcinoma (HeLa) cell proliferation.

**Methods:**

The proliferation and apoptotic effects of HeLa cells treated with EESMS were evaluated using 3-[4,5-dimethylthiazol-2-yl]-2,5 diphenyl tetrazolium bromide assay, dual acridine orange/ethidium bromide double staining, flow cytometry, and western blotting. Single-cell level atomic force microscopy (AFM) was conducted to detect the mechanical properties of HeLa cells, and proteomics and bioinformatics methods were used to elucidate the molecular mechanisms of EESMS.

**Results:**

EESMS treatment inhibited HeLa cell proliferation by blocking the G0/G1 phase, increasing the expression of Caspase-3 and affecting its mechanical properties, and the EESMS indicated no significant inhibitory effect on mouse fibroblasts L929 cell line. In total, 218 differentially expressed proteins were identified using two-dimensional electrophoresis, and eight differentially expressed proteins were successfully identified using matrix-assisted laser desorption/ionization-time-of-flight mass spectrometry. The differentially expressed proteins were involved in various cellular and biological processes.

**Conclusion:**

This study provides a perspective on how cells change through biomechanics and a further theoretical foundation for the future application of *Sophora moorcroftiana* as a novel low-toxicity chemotherapy medication for treating human cervical cancer.

**Supplementary Information:**

The online version contains supplementary material available at 10.1186/s12906-024-04502-5.

## Background

Cervical cancer (CC) is the most common gynecological malignancy [1]. Globally in 2022, there were an estimated 661,021 cervical cancer cases and 348,189 deaths, with a corresponding age-standardised incidence of 14.1% and mortality rate of 7.1% [2]. Human cervical carcinoma (HeLa) cells, a cell line derived from human cervical carcinoma, have recently been used to explore the molecular mechanisms underlying human cervical carcinoma. For example, Wang et al. found that microRNA-155-5p inhibits the migration and invasion of CC cells by inhibiting pyruvate dehydrogenase kinase 1 expression [3]. However, Fan et al. discovered that long non-coding RNA PTEN pseudogene-1 promotes the aggressive behaviors of cervical carcinoma by regulating the microRNA-106b signaling axis [4]. Therefore, exploring new, low-toxicity, and high-efficiency chemotherapeutic drugs for treating CC and improving its prognosis is essential.

The ethanol extract of *Sophora moorcroftiana* seeds (EESMS), an endemic shrub in Tibet, China, Bhutan, N Burma, and N India, is a type of Leguminous Locust found in wide valleys and on semi-arid mountain slopes from 2,800 to 4,400 m above sea level along the middle reaches of the Yarlung Zangbo River. The seed is the most valuable part of the plant, and its active ingredients are mainly matrine alkaloids and flavonoids [5, 6], which has therapeutic effect on many kinds of tumors, as shown in Table S1. *Sophora moorcroftiana* has antibacterial, immunoregulatory, anti-parasitic, anti-aging, and other effects [7–10]. The aqueous extract from the seeds of *Sophora moorcroftiana* can inhibit tumor growth and prolong the survival time of tumor-bearing mice [11]. Furthermore, flavonoids extracted from *Sophora moorcroftiana* exhibit tumor-specific cytotoxic effects [12]. Additionally, EESMS inhibits the proliferation of the human hepatoma cell line HepG2 [13], whereas 95% EESMS can inhibit proliferation and induce apoptosis in gastric cancer cells SGC-7901 [14].

Currently, research on the antitumor effect of *Sophora moorcroftiana* primarily focuses on the functional level of *Sophora moorcroftiana* alkaloids, which inhibit the proliferation of carcinoma cells, induce cell apoptosis, and cause cell cycle arrest [13]. However, the effect of *Sophora moorcroftiana* alkaloids on human cervical carcinoma has not been reported, and its antitumor mechanism has seldom been investigated. Furthermore, *Sophora moorcroftiana* is typically used as a living fuel and animal feed, and its medicinal value remains unclear, resulting in significant waste. Therefore, in this study, 3-[4,5-dimethylthiazol-2-yl]-2,5 diphenyl tetrazolium bromide (MTT) assay, dual acridine orange/ethidium bromide (AO/EB) double staining method, flow cytometry, and single-cell level atomic force microscopy (AFM) were used to detect the effect of EESMS on HeLa apoptosis, and the anti-tumor mechanism of EESMS was preliminarily explored using proteomics and bioinformatics methods. This study presents viewpoints for discovering more low-toxicity and effective chemotherapeutic drugs for CC. In addition, it can provide an experimental basis for developing and using the essential medicinal resource of *Sophora moorcroftiana.*

## Materials and methods

### Preparation of EESMS

The seeds of *Sophora moorcroftiana* were collected from the middle reaches of the Yarlung Tsangpo River, Tibet, and air-dried at room temperature (RT) for 3 weeks. Professor Hongyu Li (School of Pharmacy, Lanzhou University, Lanzhou, China) identified the seeds. A herbarium voucher specimen of the seeds (M2001-1005) was deposited in the Department of Immunology of Lanzhou University. Professor Xingming Ma (School of Basic Medicine, Lanzhou University) provided the 95% EESMS, and we got written permission to use it by Professor Ma. The extraction process was as follows. The 95% EESMS was prepared by dissolving the seeds in 4,000 mL of 95% ethanol solution and soaked at RT for 5 d before being extracted using a reflux method at 80 °C for 4 h three times successively, followed by filtration and concentration in vacuum. The resulting EESMS was then dissolved in a cell culture medium at the concentrations (1.0, 2.0, and 4.0 µg/µL) required for the experiments.

### Source of cell lines

The HeLa cell line was donated by the Center of Biomedical Experimental Research at the Medical School, Lanzhou University (Lanzhou, China), and identified as the HeLa cell using short tandem repeats. The cells were cultured in Dulbecco’s modified Eagle’s medium (Gibco, Beijing, China) supplemented with 10% fetal bovine serum (BioInd, Beit HaEmek, Israel), 100 U/mL penicillin and 100 g/mL streptomycin (Hyclone, Utah, USA) at 37 ℃ in incubator with a humidified atmosphere of 5% CO_2_ and 95% air. As well, L929 cells obtained from the American Type Culture Collection (ATCC, CCL-1, USA) were cultured in Eagle’s minimum essential medium (Gibco, Beijing, China) containing 10% newborn calf serum (NCS) (Bovogen, China) and 5000 U/mL penicillin–streptomycin (Thermo Scientific, USA) in a 5% CO_2_ incubator at 37 ℃. Cells in the mid-log phase were used in the experiments.

### MTT testing

HeLa cells were inoculated into 96-well plates overnight at a concentration of 2 × 10^3^ cells/mL. After the cells had attached to the wall, 200 µL of drugs with different concentrations were diluted in a complete medium and added to the experimental group with an equal volume of complete medium. The culture was terminated after 36 h, and 20 µL MTT (5 mg/mL) solution was added to each hole. The samples were then incubated for 4 h without light. The supernatant was discarded, and 150 µL of dimethylsulfoxide solution was added to each hole and shaken at low speed for 10 min at RT. The optical density (A) at a wavelength of 570 nm was determined using an enzyme-labeling instrument, and the relative and half inhibition rates of cell growth were calculated. This process was repeated thrice. The half-maximal inhibitory concentration (IC_50_) value was subsequently used to treat HeLa cells using flow cytometry and two-dimensional gel electrophoresis (2-DE) analysis. The cell growth inhibition rate was calculated using the following formula: Cell growth inhibition rate = (A control group - A test group) / (A control group - A blank group) × 100%.

### CCK-8 assay

The effect of EESMS on the activity of mouse fibroblasts L929 cell line was tested by CCK-8 cell proliferation-toxicity kit. L929 cells were seeded in 96-well plates at a density of 5000 cells/well and cultured at 37 ℃ for 12 h. Then the cells were treated with the ethanolic extracts at different concentrations (0, 1, 2, 4 µg/µL), and the culture medium was discarded after 1, 2, and 3 days. After washing with PBS twice, 100 µL of CCK-8 working solution was added, and incubated at 37 ℃ for 1 h. Optical density of each well was detected by an enzyme-labeling instrument at 450 nm.

### AO/EB dyeing and observation

HeLa cells were seeded at a density of 7 × 10^4^/holes into 12-well plates. After 24 h of incubation, the cells were attached, and the original medium was discarded. Different drug concentrations were added to the experimental group (1.0, 2.0, and 4.0 µg/µL), and the negative control group was incubated in a cell incubator with the same volume of complete medium. The medium was discarded at 36 h, and the cells were washed twice with phosphate-buffered saline (PBS) to remove the residual medium and unadherent cells. Furthermore, 2 mL PBS and 10 µL AO/EB dye (100 µg/mL) were added to each hole for 2 min at RT for staining. The cells were then placed under a fluorescence microscope to observe cell morphology and to identify living cells, viable apoptotic cells, non-viable apoptotic cells (NVA), and necrotizing cells based on morphological characteristics, such as cell size, nucleoplasm ratio, nuclear coloration, nuclear shape, and apoptotic bodies. The experiment was repeated thrice.

### Single-cell level AFM

A biological AFM (BioAFM; NanoWizard III, Bruker, Germany) was used for all mechanical performance analyses. The uniqprobe™ BioAC series of silicon AFM cantilevers with a force constant of 0.06 N/m were used (qp-BioAC-CI, NanoSensors, Switzerland). The cells attached to the round cover glass were placed in a Biocell (JPK Instruments, Germany), the AFM tip was positioned in the cell area, and the cells to be imaged were selected for a backward scan. The contact mode was used, the image resolution was 512 × 512 pixels, and the tip scanning rate was 0.25 Hz. The morphology and mechanical properties of the sample were measured at 5 μm/s as the probe touched the cells, generating a force-distance curve. Six random sites were selected for each sample, and each site was measured 15 times. We then calculated the mechanical characteristics of Young’s modulus and roughness.

### Cell cycle analysis

HeLa cells were collected and inoculated into a culture bottle at a concentration of 1 × 10^5^ cells/mL overnight. Furthermore, different EESMS concentrations were added to the experimental group (1.0, 2.0, and 4.0 µg/µL), whereas the control group was provided the same amount of complete culture medium and trained for 36 h. The cells were digested with ethylenediaminetetraacetic acid (EDTA)-free trypsin, washed twice with PBS, and centrifuged for 5 min at 1000 r/min. The original medium was discarded. Furthermore, 70% cold ethanol was added, and the cells were placed overnight in a refrigerator at 4 ℃. The cells were digested with RNA enzymes and stained with propidium iodide (PI) for 30 min in the dark. The processed samples were analyzed using flow cytometry (BD Biosciences, China), and the data were statistically analyzed. This process was repeated three times.

### Apoptosis analysis

The cells were allowed to attach to the wall after different EESMS concentrations were added to the experimental group (1.0, 2.0, and 4.0 µg/µL). However, the control group was provided the same amount of complete culture medium and trained for 36 h. The cells were digested with EDTA-free trypsin, and the concentration of cells was adjusted to 1 × 10^6^ /mL. Furthermore, 1 mL of cells were centrifuged for 10 min at 1000 r/min and 4 ℃. The original medium was discarded, and the cells were washed thrice with 1 mL of pre-cooled PBS.

Furthermore, the cells were centrifuged again for 10 min at 1000 r/min and 4 ℃, and the original medium was discarded. The cells were then suspended in 200 µL of binding buffer. The cells were blown, mixed, and incubated for 15 min at RT without light after adding 10 µL of Annexin V-fluorescein isothiocyanate and 10 µL of PI. In addition, 300 µL binding buffer was added and immediately detected using flow cytometry.

Each group of HeLa cells was treated with different EESMS concentrations, followed by the harvest of adherent and floating cells after 36 h. Total cellular proteins were extracted by incubating with the radioimmunoprecipitation assay buffer (Sigma-Aldrich, MO, USA) for 15 min and centrifuged at 13, 500 rpm for 30 min at 4 ℃. Furthermore, the bicinchoninic acid assay was used to determine the protein concentrations. The 20 µg protein extracts obtained from each group were transferred onto polyvinylidene fluoride membranes (Beijing Solarbio Science & Technology Co., Ltd., China) after separating them into 10–12% sodium dodecyl sulfate and completing the polyacrylamide gel electrophoresis. They were then blocked with 5% milk in Tris-buffered saline (GTX48883, GeneTex, China) and Tween-20 (Sigma-Aldrich, St. Louis, MO, USA) for 1 h. Furthermore, the membranes were incubated with primary antibodies against Caspase-3 (1:500, Beyotime Biotechnology, China) at 4 ℃, followed by incubation with the secondary antibody (1:5000; Thermo Fisher Scientific, China) for 1 h. The immobilized protein bands were visualized using an enhanced chemiluminescence kit (Millipore Corporation, Billerica, MA, USA). A BioSpectrum Imaging System (Analytik Jena US LLC, Upland, CA, USA) was used to capture band images. The ImageJ software 1.8.0_172 (National Institutes of Health, USA) was used to quantify the relative densitometry of the immunoreactive bands. Glyceraldehyde 3-phosphate dehydrogenase was used as a loading control, and the data obtained were statistically analyzed.

### Extraction of total cell protein

The cells were collected and inoculated in a culture bottle at a concentration of 1 × 10^4^ cells/mL overnight. An EESMS of 2 µg/µL was added to the experimental group, whereas the control group was provided the same amount of complete culture medium. Cells were then trained for 36 h. Trypsin (0.25%) was added to the cells and incubated for 1 min, and 2 mL of the complete medium was added to stop digestion. The cells were centrifuged at 1,000 r/min for 5 min, and the original medium was discarded. The cells were washed thrice with 1 mL of pre-cooled PBS, blown, and mixed to suspend the cells. The mixture was centrifuged for 5 min at 1,000 rpm, and the original medium was discarded. The cell lysate (200 µL cell lysate per 2 × 10^6^ cells) was added. The mixture was allowed to stand still for 30 min at 4 ℃, centrifuged for 1 h at 16,000 r/min and 4 ℃, and the original medium was collected and stored in a refrigerator at -80 ℃.

### Determination of protein concentration using the Bradford method

Bovine serum albumin (BSA) standard solutions of 0, 1, 2, 3, 4, 5, and 6 µL were added to the second row of 96-well plates, 1 µL from the experimental and control groups were added to the ninth and tenth holes respectively, and the total protein samples to be tested were added to PBS and supplemented to 15 µL. Each group contained six holes. Furthermore, 285 µL was added to each hole and gently blown and mixed. The mixture was set still for 5 min. Absorbance values at 595 nm were determined using an enzyme-labeling instrument. The absorbance values of the unadded BSA group were used as blank controls. The standard curve for BSA was made in CurvExpert Pro 2.6.5, and the experimental and control groups were calculated using the obtained formula for the protein concentration of group samples.

### Isoelectric focusing

A tube of hydrated sample buffer (1 mL in separate, -20 ℃ frozen, free of dithiothreitol and amphoteric electrolyte) was obtained and statically dissolved. Dithiothreitol (0.01 g) was mixed into the removed tubules, and 5 µL of bio-lyte was prepared in advance and frozen. The control and experimental groups were provided appropriate amounts of samples (calculated based on the sample’s protein concentration so that the protein sample amount was 80 µg), and the hydration sample buffer solution in the above tubules was replenished to 300 µL and mixed well. The frozen, immobilized pH gradient-preformed strips (17 cm, pH 3–10, non-linear) were thawed, and the samples were blown, remixed, and added to the focusing plate to ensure the consistency of the sample liquid to avoid bubble formation. The plastic protective film on the rubber strip was removed, and the rubber strip was placed on the sample solution with the rubber surface in contact with the sample liquid facing downward and the positive electrode of the rubber strip in relative and close contact with the positive electrode of the focusing disc. Furthermore, 1.5 mL of mineral oil was taken and slowly added dropwise from left to right onto the plastic supporting film to avoid the evaporation of the sample liquid. Isoelectric focusing and balancing were performed immediately.

### Sodium dodecyl sulfate-polyacrylamide gel electrophoresis cataphoresis

Two 10% acrylamide gel electrophoreses were prepared. The residual mineral oil and excess sample were gently absorbed from the rubber strip on a thick, wet filter paper. The rubber strip was transferred to the sample hydration plate for the first and second balances, and the excess balance solution was absorbed with a soaked thick filter paper. The filled gel was placed correctly, a dissolved agarose sealant solution and 1 × electrophoresis buffer were prepared, and the rubber strip was immersed in 1× electrophoresis buffer for washing. The adhesive tape was placed face up on the long glass plate of the gel, and an agarose sealant solution was added dropwise to the gel. The tape was gently pushed down with the needle of the flat head so that the lower end of the tape was in full contact with the separating glue surface, and all the bubbles were discharged. After 10 min, the agarose gel solution was completely solidified, and the gel was transferred to an electrophoresis tank for sodium dodecyl sulfate-polyacrylamide gel electrophoresis. After electrophoresis, the gel was removed, and the positive electrode was marked with an angle, cut, and stained. The staining was performed using a Fast Silver Dyeing Kit (P0017S, Beyotime Biotechnology, China).

### Gel scanning

After staining, the gel was placed in a VersaDoc 4000 imaging system with a high-resolution scanner to capture images. Image analysis software (PDQuest Advanced 8.0.1) was used to scan the gel map (resolution of 300 PPI) in a greyscale. Analysis software was used to modify the gel map, shear, protein point detection optimization, background subtraction, and re-matching establishment. The gel images were analyzed in combination with manual correction. A statistical data analysis was performed if the relative variation of protein points in the map was greater than twice the difference in protein points, and the *t*-test was used between groups.

### Mass spectrometry identification

The 10 differentially expressed proteins were selected from the gels of the control group. After enzymatic hydrolysis, an extraction API 4800 tandem time-of-flight mass spectrometer (MALDI-TOF; Applied Biosystems) was used for enzymatic hydrolysis, extraction, and sample preparation. The parameter settings were as follows: positive ion mode automatically acquired data. The peptide mass fingerprinting mass spectrometry scan range was 800–3500 Da, and the 10 peaks with the highest intensity were selected for secondary mass spectrometry. GPS 3.6 (Applied Biosystems) was applied after the accurate molecular weight determination of the peptide fragments, and Mascot 2.1 (Matrix Science) software was used to analyze and identify the integrated primary and secondary mass spectrometry data.

### Bioinformatics analysis

The PDQuest 8.01 software was used to pair and match the protein points between the experimental and control groups to create a qualitative and quantitative analysis dataset, and 218 different protein points were found after a comprehensive comparison. From the control group, 10 differentially expressed protein points (the difference was more than twice, and the same in the gel atlas of the three groups) were selected for MALDI-TOF mass spectrometry identification, and eight proteins were successfully identified.

### Statistical analysis

All data were analyzed using IBM SPSS software (version 26.0). Data were expressed as mean ± standard deviation, the differences between groups were analyzed using a *t*-test (two groups) and one-way analysis of variance (three or more groups). Following, we performed LSD analysis for multiple comparisons. Statistical significance was set at *P* < 0.05.

## Results

### Inhibitory effect of EESMS on HeLa cell proliferation

**Fig. 1:**
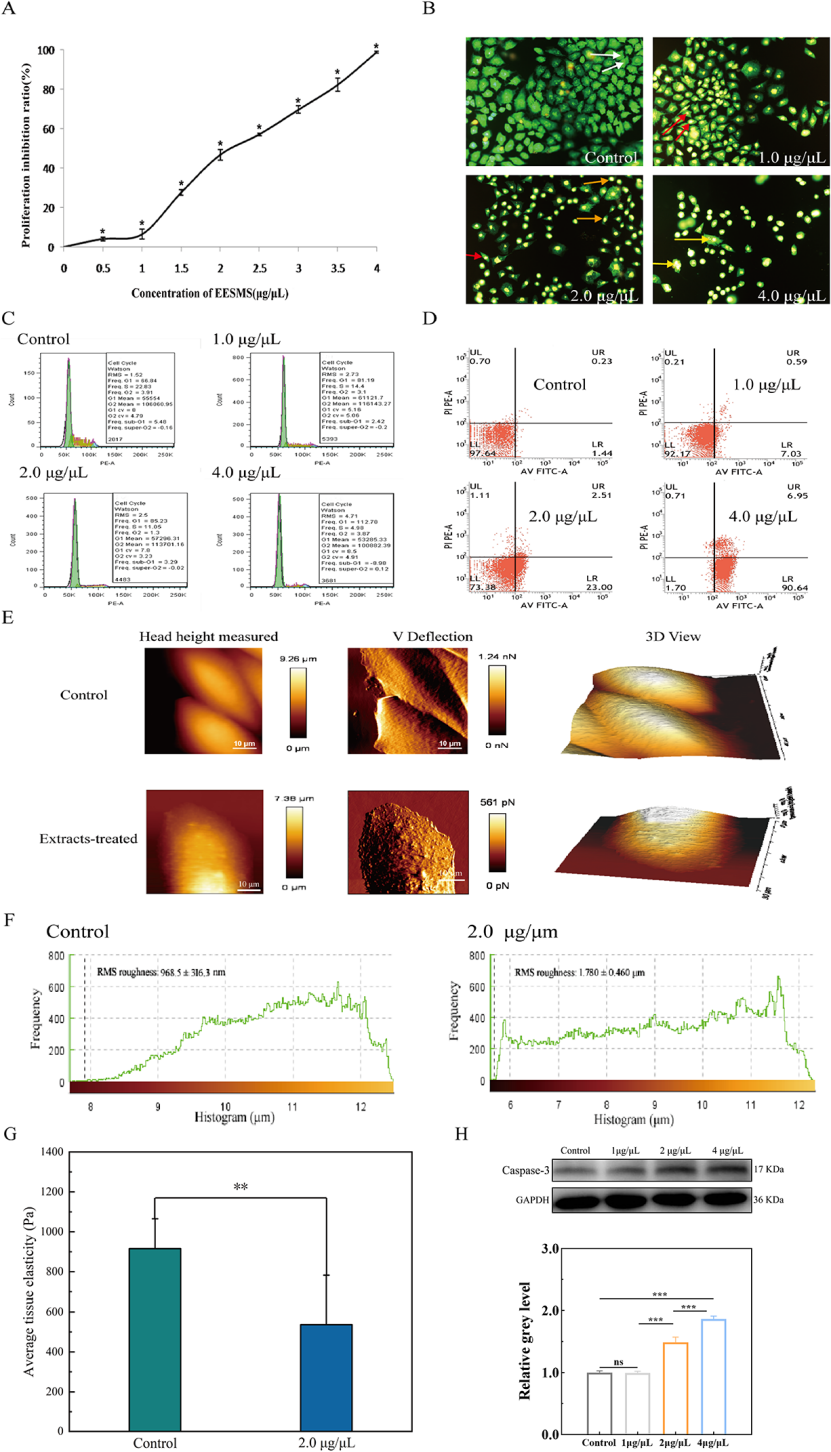
The growth-inhibitory effects of EESMS on Hela cell. **A**: Inhibition of Hela cell proliferation by different concentrations of EESMS. **B**: Apoptosis of Hela cell by AO/EB staining in different dose groups. White arrow was VN; Red arrow was VA; Orange arrow was NVA; Yellow arrow was NVN. **C**: Cell cycle changes of Hela treated with EESMS at different concentrations. **D**: Apoptosis rate of Hela cells by different concentrations of EESMS. **E**: AFM imaging about the morphology of Hela cells in the control and 2.0 µg/µl group. **F**: Cell surface roughness in the control and 2.0 µg/µl group. **G**: Average cell elasticity in the control and 2.0 µg/µL group. **H**: Western blot results and relative gray level of each group

MTT assay showed that EESMS inhibited the proliferation of HeLa cells dose-dependently (Fig. 1A). The drug dose was sequentially increased in the experimental group. The growth-inhibitory rates were 4.12 ± 0.94%, 6.57 ± 2.49%, 27.69 ± 1.32%, 46.77 ± 2.74%, 57.07 ± 0.69%, 69.74 ± 1.94%, 82.29 ± 3.37%, and 98.71 ± 0.46%, compared with untreated controls, and this was considered to indicate a statistically significant difference (*P*<0.05). The IC_50_ of the ethanolic extracts for HeLa cells was 2.085 µg/µL.

In addition, the effect of ethanolic extracts from *Sophora Moorcroftiana* seeds on the activity of mouse fibroblasts L929 cells line was tested by CCK-8 cell proliferation-toxicity kit. The results of CCK-8 assay showed that, with the increase of time and concentration, the extract of *Sophora Moorcroftiana* seeds indicated no significant inhibitory effect on mouse fibroblasts L929 cell line. As shown in Figure S1.

### Apoptotic morphology of EESMS-induced HeLa cells

The observation of the cells under a fluorescence microscope revealed no significant apoptotic cells in the controls. In control cells, cell morphology was normal, the cytoplasm was plump, and there was no pyknosis in the nucleus, which was green with a normal structure. Early apoptotic cells were observed in the 1.0 µg/µL dose group, and the cell nuclear chromatin was green with pyknotic or lumpy shape, lying on one side of the cell. The number of early and late apoptotic cells increased with increased EESMS dose, and the morphology of the NVA changed. The cytoplasm and nucleus were condensed, and the nucleocytoplasmic ratio was inverted. The cell nucleus chromatin was orange, compact, heavily stained, and inclined toward one side of the cell, and apoptotic bodies were observed in some cells. The chromatin of the dead cell nuclei was orange and indistinct. With increased drug concentration, the number of apoptotic and necrotic cells increased, and this number was positively correlated with the drug concentration (Fig. 1B).

### Effect of EESMS on HeLa cell cycle

Flow cytometry results indicated that EESMS significantly blocked the HeLa cell cycle and blocked cell division in the G0/G1 phase. The proportion of cells in the S and G2/M phases gradually decreased with increasing drug concentration, whereas the proportion of cells in the G0/G1 phase increased significantly. Notably, the representation showed an obvious concentration dependence, and the difference between each group was statistically significant compared with that in the controls (*P* < 0.05) (Table 1; Fig. 1C).

**Table 1:** The changes of Hela cell cycle after EESMS treatment

Concentration	Cell cycle ratio		
	G0/G1	S	G2/M
0 ug/ul	64.23 ± 7.45	24.19 ± 1.93	5.58 ± 0.81
1 ug/ul	76.46 ± 9.79^#^	15.22 ± 2.06^#^	7.32 ± 1.03^#^
2 ug/ul	83.48 ± 10.69^#^	9.72 ± 1.36^#^	5.80 ± 0.69^#^
4 ug/ul	91.67 ± 17.17^#^	5.71 ± 1.12^#^	2.62 ± 0.71^#^
P value	0.014	0.007	0.02

*Note*^#^*P*<0.05 vs. Control (0 ug/ul)

### Effect of EESMS on HeLa cell apoptosis

Flow cytometry showed that the early apoptosis rate of the experimental group was significantly different compared with that in the control group. The early apoptosis rate of cells in the experimental group gradually increased with increased EESMS concentrations, showing an obvious concentration correlation. Moreover, compared with the control group, the late apoptosis rate in the experimental group was also significantly different, and the late apoptosis rate showed an obvious concentration dependence (Table 2; Fig. 1D). Western blotting results demonstrated that the expression of Caspase-3 was higher in the experimental groups than that in the control and increased with increasing EESMS concentrations (Fig. 1H).

**Table 2:** The effects of EESMS on apoptosis rate in Hela cells

Concentration	Apoptosis rate		Necrosis rate
	Early apoptosis rate	Late apoptosis rate	
0 ug/ul	1.57 ± 0.41	0.28 ± 0.15	0.52 ± 0.29
1 ug/ul	7.11 ± 1.04^#^	0.64 ± 0.06^#^	0.32 ± 0.13^※^
2 ug/ul	22.74 ± 6.69^#^	2.72 ± 0.88^#^	2.46 ± 0.59^#^
4 ug/ul	83.29 ± 11.35^#^	7.71 ± 2.09^#^	0.89 ± 0.44^※^
P value	0.001	0.02	0.06

*Note*^#^*P*<0.05 vs. Control (0 µg/µl), ^※^*P*>0.05 vs. Control

### Changes in mechanical characteristics caused by EESMS

The morphology of HeLa cells in the control and experimental groups was investigated using biological AFM (Fig. 1E). As shown in Fig. 1E, the two head height measured pictures showed the 50 μm×50 μm morphologies of HeLa cells in the control and experimental groups. The two middle pictures show the corresponding deflections and the last two pictures show the corresponding three-dimensional images. HeLa cells grew well with clear contours and irregular polygonal structures, and there were many whiskers around the cells, showing the surface characteristics of tumor cells. Figure 1F shows that the average range of cell elasticity was 200–1600 Pa. In the control group, higher stiffness was observed than in the 2.0 µg/µL group (*P* < 0.05). It can be inferred that HeLa cells were released after the EESMS treatment, leading to a decrease in the average cell elasticity of the HeLa cells. Figure 1G summarizes the surface roughness values of the experimental and control groups. The mean surface roughness of cells in the 2.0 µg/µL group was 1.780 ± 0.460 μm, while that in the control group was 968.5 ± 316.3 nm. Therefore, after EESMS treatment, the number of HeLa cells was significantly reduced, with rough surfaces and approximate regularity, indicating that EESMS inhibited the growth of HeLa cells.

### Total protein concentration determined using the Bradford method

**Fig. 2:**
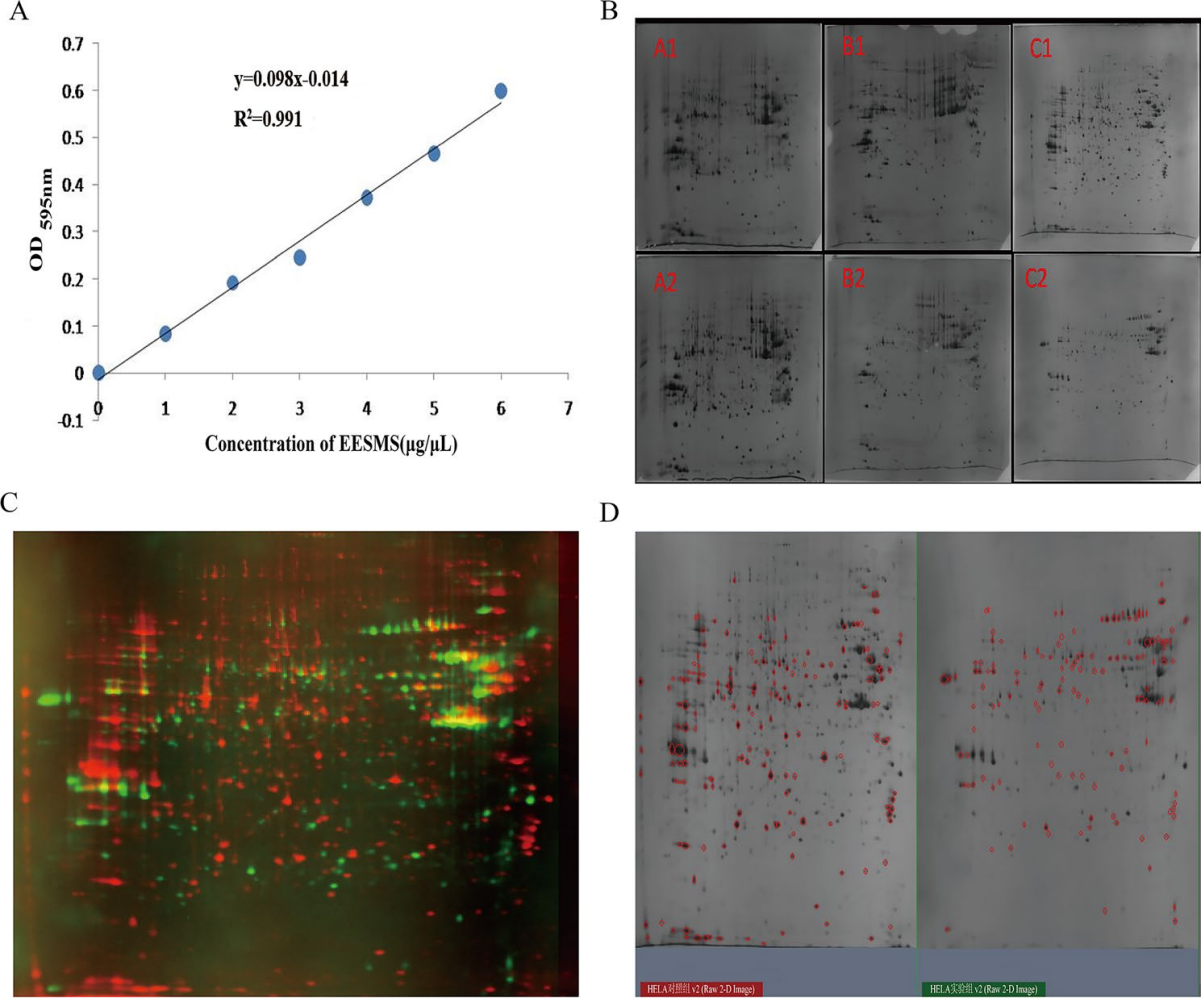
The 2-DE maps of control and EESMS-treated Hela cell. **A**: The standard curve of protein concentration by Bradford method. **B**: Gel pattern by three two- dimensional electrophoresis experiments (*Note* the pictures A1, B1 and C1 were respectively the gel graphs of the control for three times, A2, B2 and C2 were respectively the gel graphs of the 2.0 µg/µL EESMS). **C**: Gel consistency matching map between experimental group and control group (Note: Protein points were labeled in red for the control group and in green for the experimental group, and the higher the coincidence degree of two colors of the same protein point, the stronger the consistency and matching degree). **D**: Comparison and analysis of protein maps between experimental group and control group (0 µg/µL Control, 2.0 µg/µL EESMS Group) (*Note* The red marks indicated points that are more than twice as different, called differential protein points, 218 in all)

Total protein was extracted from cells of the experimental and control groups. The Bradford method was used to detect the protein concentration, and a standard curve was drawn. The total protein concentration in each sample was calculated using a standard curve formula (Fig. 2A).

### Two-dimensional electrophoretic pattern and consistent matching results

The total protein content of the samples was subjected to 2-DE, and the experiment was repeated thrice. PDQuest8.01 software was used to analyze the obtained 2-DE gel pattern. The chromatograms obtained from three experiments were colored, replaced, and overlapped. The better the matching of common protein points between the two groups, the higher the degree of coincidence. The non-overlapping points represent different protein points between the experimental and control groups. Furthermore, the most reproducible atlas was selected for further analysis (Fig. 2B and C).

### Analysis of differential protein points in two-dimensional electrophoresis gel pattern

The PDQuest 8.01 software matched the different protein points and data between the experimental and control groups with the best repeatability. In total, 218 protein points were identified. In addition, different protein points were compared again through visual observation, and 10 different protein points were selected for further mass spectrometry identification (Fig. 2D).

### Selection of differential protein spectrum identification results

**Fig. 3:**
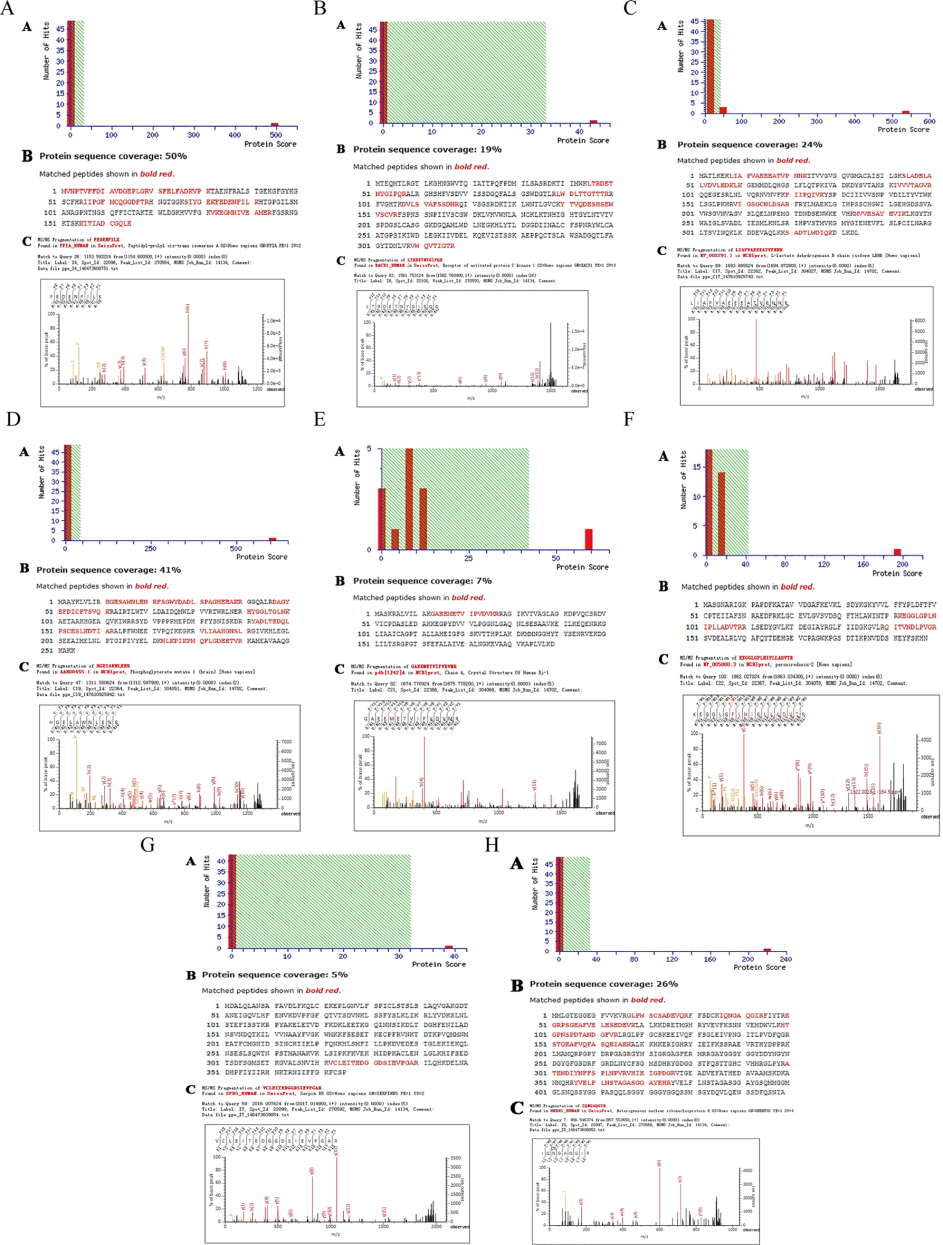
Representative MALDI-TOF/TOF MS maps and database query results. **A**: PPIA mass spectrometry identification results. **B**: RACK1 mass spectrometry identification results. **C**: LDHB mass spectrometry identification results. **D**: PGAM1 mass spectrometry identification results. **E**: PARK7 mass spectrometry identification results. **F**: PRDX2 mass spectrometry identification results. **G**: SERPIN B5 mass spectrometry identification results. **H**: HNRNPH1 mass spectrometry identification results. (**A**: Mascot score histogram, **B**: Amino acid sequence, marked in red as the matching sequence, **C**: Partial peptide map)

The 10 differential protein points in the control group with upregulated expression and in the experimental group with downregulated expression were sent to Shanghai Shenggong Company for MALDI-TOF mass spectrometry detection, and eight differential protein points were successfully identified. They were peptidyl-prolyl cis-trans isomerase A (PPIA/CypA), receptor of activated protein C kinase 1 (RACK1), L-lactate dehydrogenase B chain isoform (LDHB), phosphoglycerate mutase 1 (PGAM1), chain A, crystal structure of human Dj-1 (PARK7), peroxiredoxin-2 (PRDX2), serpin B5 (SERPINB5), and heterogeneous nuclear ribonucleoprotein H-1 (HNRNPH1) (Table 3; Fig. 3).

**Table 3:** Identification results of MALDI-TOF/TOF-MS

Protein name	Gene name	UniProtKB no.	Calcul- ated PI	Nominal mass (Mr)	Mascot score
Peptidyl-prolylcis-trans isomerase A	PPIA	P62937	7.68	18,229	497
Receptor of activated protein C kinase 1	RACK1	P63244	7.60	35,511	43
L-lactate dehydrogenase B chain isoform	LDHB	P07195	5.71	36,900	535
Phosphoglycerate mutase 1	PGAM1	P18669	6.67	28,888	606
Chain A, Crystal Structure Of Human Dj-1	PARK7	Q99497	6.33	20,063	59
Peroxiredoxin-2	PRDX2	P32119	5.66	22,049	195
Serpin B5	SERPINB5	P36952	5.72	42,530	39
Heterogeneous nuclear ribonucleoprotein H1	HNRNPH1	P31943	5.89	49,484	220

### Bioinformatics analysis of differential proteins

**Fig. 4:**
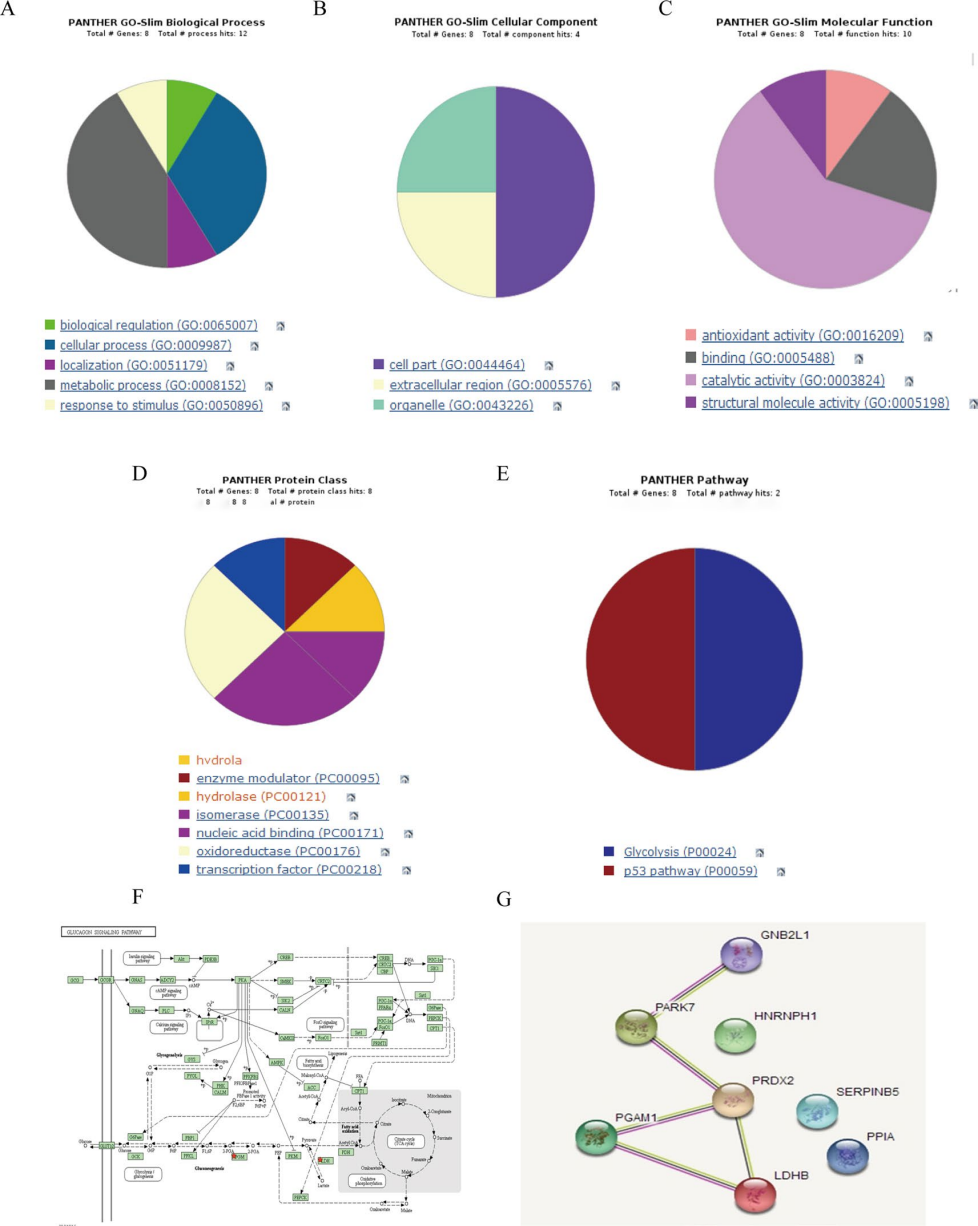
Bioinformatics analysis. **A**: BP analysis, **B**: CC analysis, **C**: MF analysis, **D**: PC analysis, **E**: PP analysis, **F**: Partial protein KEGG signal pathway, **G**: String toolkit showed the interactions between eight different proteins (*Note* The blue line represents the co-expression relationship, the purple line represents experimental verification, the yellow lines represent evidence of text mining)

The STRING database (http://string-db.org/) was used to analyze the interaction information of the eight differentially expressed proteins. Gene Ontology (http://www.g-eneontology.org/) analysis of the differentially expressed proteins was performed using the DAVID (http://david.abcc.ncifcrf.gov/) or PANTHER (http://www.pantherdb.org/) databases. We analyzed the proteins involved in biological processes, cellular components, molecular functions, protein classes, and other pathways (PP). The Kyoto Encyclopedia of Genes and Genomes database (http://www.genome.ad.jp/kegg/pathway.html) and other databases were used to analyze the signal pathway of differential proteins. The results showed that the biological processes involved in the differential proteins mainly included oxidative stress response, negative regulation of gene expression, cell adhesion, negative regulation of apoptosis induction, oxidation-reduction (REDOX) processes, and positive regulation of protein secretion (Table 4; Fig. 4).

**Table 4:** Gene otology analysis

Category	Term	Genes
Biological Process	positive regulation of protein secretion	PPIA
	cellular response to oxidative stress	PARK7/PRDX2
	negative regulation of gene expression	PARK7/RACK1
	cell-cell adhesion	PARK7/RACK1
	negative regulation of apoptotic process	PARK7/PRDX2
	oxidation-reduction process	LDHB/PRDX2
Cellular Component	myelin sheath	LDHB/PGAM1
	membrane raft	LDHB/PARK7
	mitochondrion	LDHB/PARK7/RACK1
	extracellular exosome	PPIA/RACK1/LDHB PGAM1/ PARK7 PRDX2/ SERPINB5
	cytoplasm	RACK1/LDHB/PARK7 PGAM1/ HNRNPH1 PRDX2/SERPINB5
	cytosol	PPIA/RACK1/LDHB PGAM1/PARK7/PRDX2
	membrane	PPIA/LDHB PGAM1/HNRNPH1
Molecular Function	kinase binding	LDHB/PARK7
	poly(A) RNA binding	RACK1/HNRNPH1/PPIA
	cadherin binding involved in cell-cell adhesion	RACK1/ PARK7
	enzyme binding	RACK1/ PARK7
	receptor binding	RACK1 /PARK7
	protein homodimerization activity	RACK1/ PARK7
	identical protein binding	PARK7/LDHB
	protein binding	PPIA/RACK1/LDHB PGAM1/PARK7 SERPINB5/HNRNPH1

## Discussion

CC is the most common gynecological malignancy. It is characterized by a poor prognosis and a low survival rate. HeLa cells are one of the cell lines in human cervical carcinoma. Therefore, to develop novel and low-toxicity therapeutic strategies for CC, it is essential to understand the underlying mechanisms of the growth, invasion, and metastasis of HeLa cells.

The cell proliferation assay results showed that EESMS could inhibit the proliferation of HeLa cells dose-dependently, with an IC_50_ of 2.085 µg/µL. Cell cycle analysis showed that the proportion of apoptotic cells increased in the early and late stages, and the cell cycle was blocked in the G0/G1 phase after EESMS treatment. EESMS induced apoptosis in HeLa cells and the number of apoptotic cells was positively correlated with the concentration of EESMS from different perspectives. Morphological observations using AO/EB double staining showed that early apoptotic cells appeared in the low-concentration EESMS group (1.0 µg/µL). The number of late apoptotic cells gradually increased with increased drug concentration, and a few necrotic cells were observed. Conventional apoptosis was determined using western blotting, which revealed that Caspase-3 expression increased with increasing EESMS concentrations.

The actin cytoskeleton, the key structure of the cell, dynamically combines and rearranges to support various cellular behaviors, including growth, proliferation, migration, and death [15, 16]. Notably, some aspects of the biomechanical findings have affirmed that elasticity can reflect the hardness of malignant cells or tissues, indicating changes in cancerous tissues [17]. In this study, the results of single-cell mechanics suggested that EESMS treatment of HeLa cells led to decreased elasticity, which could be attributed to increased Caspase-3 activity. This increased activity may be responsible for the cleavage of cytoskeletal actin and gelsolin, resulting in F-actin depolymerization during apoptosis [18–20]. Furthermore, an increasing roughness on the cell surface was observed in the 2.0 µg/µL ESSMS group, which may potentially indicate cancer progression. Biomechanical methods can provide supplementary means for determining tumor cell phenotypes.

Furthermore, 2-DE was utilized to explore the mechanism through the proteomic study of the total proteins of HeLa cells in the control and EESMS testing groups, and eight kinds of differentially expressed proteins were selected. These differentially expressed proteins were upregulated in the control group and downregulated in the experimental group and were analyzed in detail with related literature and bioinformatics analysis. PPIA, Cyclophile A (Cyclophilin A, CypA), catalyzes the formation of active space structures of proteins containing proline and plays a key rate-limiting role in protein folding and assembly. Notably, several studies have shown that the level of RACK1 expression is positively correlated with the proliferation, growth, infiltration, and invasion of many kinds of tumor cells. RACK1 can bind to cyclic adenosine monophosphate, AKT, Src, stratified proteins, and other molecules to regulate tumor cell growth, proliferation, migration, and the cell cycle through multiple signaling pathways. Lactate dehydrogenase is a key enzyme in glycolysis. LDHB significantly correlates with the occurrence, development, lymph node metastasis, and tumor cell proliferation in breast, lung, esophageal, bladder, and oral cancers and other malignant tumors [21–24]. Phosphoglycerate mutase is located in the middle reaches of the glycolysis pathway. It is not a rate-limiting enzyme; however, it is crucial in metabolic networks. Notably, PGAM1 is highly expressed in many types of tumors, such as liver, lung, breast, and colon cancers and glioblastoma. The activity of the PGAM1 enzyme in these tumors is higher than that in normal cells [25–27]. Dj-1 is the third gene associated with familial Parkinson’s disease [28]. This gene is a new oncogene with antioxidant, anti-apoptotic, antagonistic tumor suppressor, antitumor properties, and other functions [29–34]. PRDX2 exists in eukaryotic cells and is widely expressed in various tissues. According to the literature, PRDX2 is highly expressed in head and neck cancers [35], colon cancer [36], anterior adenocarcinoma [37], and osteosarcoma [38]. However, it is downregulated in malignant tumors, such as gastric cancer [39] and malignant melanoma [40]. Serpin B5 is a member of the Serpin family of serine protease inhibitors, also known as Maspin.

Maspin is a tumor suppressor gene [41–47]. Its expression is regulated by epigenetic modifications, such as methylation and acetylation, and transcription factors, such as p53 and p63. However, recent experimental results have shown that Maspin is highly expressed in some malignant tumors and participates in and promotes the occurrence and development of malignant tumors. In this study, Serpin B5 expression was upregulated in the control group and downregulated in the experimental group. B5 has been speculated to act as an oncogene. HNRNPH1 is a member of the nuclear heterogeneous ribonucleoprotein family, which is closely associated with the processing of precursor messenger RNA (mRNA), participates in mRNA transport and post-transcriptional regulation, and is crucial in regulating the proliferation and apoptosis of cancer cells. Therefore, the EESMS-induced apoptosis of HeLa cells may be achieved by downregulating the expression of these proteins.

In summary, EESMS induces apoptosis in HeLa cells and promotes the periodic arrest of HeLa cells in the G0/G1 phase. Based on the results of bioinformatics analysis of differentially expressed proteins and related literature reports, we speculate that this may be associated with several biological functions, such as cell oxidative stress, negative regulation of gene expression, cell adhesion, negative regulation of apoptosis induction, redox processes, and positive regulation of protein secretion. The differentially expressed proteins identified using 2-DE are a preliminary study on the EESMS-induced mechanism of apoptosis in HeLa cells, and the results of the eight differentially expressed proteins obtained need to be further verified. These consistent experimental results are probably the target proteins of the action of EESMS. Small interfering RNAs and transfection techniques can be used to modulate the expression levels of target proteins and observe the effects of these changes on cancer cell apoptosis. This supports the hypothesis that EESMS treatment of HeLa cells leads to increased apoptosis.

## Conclusions

EESMS significantly inhibited proliferation, induced apoptosis of HeLa cell lines, and blocked the cell cycle in the G0/G1 phase. The action of EESMS may be mediated through several biological functions, such as oxidative stress, negative regulation of gene expression, cell adhesion, negative regulation of apoptosis, redox processes, and positive regulation of protein secretion. This study demonstrated that EESMS positively affects human cervical carcinoma, which provided a further theoretical basis for the Tibetan medicine *Sophora moorcroftiana* as a novel low-toxicity, high-efficiency chemotherapeutic drug for treating cervical cancer in the future.

### Electronic supplementary material

Below is the link to the electronic supplementary material.

Supplementary Material 1

## Data Availability

The datasets used and/or analyzed during the current study are available from the corresponding author on reasonable request.

## Abbreviations

EESMS: Ethanol extract of *Sophora moorcroftiana* seeds
HeLa: Human cervical carcinoma
CC: Cervical cancer
MTT: 3- [4,5-dimethylthiazol-2-yl]-2,5 diphenyl tetrazolium bromide
AO/EB: Dual acridine orange/ethidium bromide
IC_50_: Half-maximal inhibitory concentration
RT: Room temperature
2-DE: Two-dimensional gel electrophoresis
PBS: Phosphate-buffered saline
NVA: Non-viable apoptotic cells
PI: Propidium iodide
BSA: Bovine serum albumin
AFM: Atomic force microscopy
mRNA: Messenger RNA

## References

[1] Arbyn M, Weiderpass E, Bruni L, Sanjose S, Saraiya M, Ferlay J, Bray F (2020). Estimates of incidence and mortality of cervical cancer in 2018: a worldwide analysis. Lancet Glob Health.

[2] Bray F, Laversanne M, Sung H, Ferlay J, Siegel RL, Soerjomataram I et al. Global cancer statistics 2022: GLOBOCAN estimates of incidence and mortality worldwide for 36 cancers in 185 countries. CA Cancer J Clin 2024. 10.3322/caac.21834 38572751

[3] Wang F, Shan S, Huo Y, Xie Z, Fang Y, Qi Z (2018). MiR-155-5p inhibits PDK1 and promotes autophagy via the mTOR pathway in cervical cancer. Int J Biochem Cell Biol.

[4] Fan Y, Sheng W, Meng Y, Cao Y, Li R (2020). LncRNA PTENP1 inhibits cervical cancer progression by suppressing miR-106b. Artif Cells Nanomed Biotechnol.

[5] Zhang Y, Mi DY, Wang J, Luo YP, Yang X, Dong S (2018). Constituent and effects of polysaccharides isolated from Sophora moorcroftiana seeds on lifespan, reproduction, stress resistance, and antimicrobial capacity in Caenorhabditis elegans. Chin J Nat Med.

[6] Shirataki Y, Motohashi N, Tani S, Sakagami H, Satoh K, Nakashima H (2001). In vitro biological activity of prenylflavanones. Anticancer Res.

[7] Wang SY, Sun ZL, Liu T, Gibbons S, Zhang WJ, Qing M (2014). Flavonoids from Sophora moorcroftiana and their synergistic antibacterial effects on MRSA. Phytother Res.

[8] Luo Y, Zhang G, Liu X, Yuan M, Gao Q, Gao H (2018). Therapeutic and immunoregulatory effects of water-soluble alkaloids E2-a from Sophora moorcroftiana seeds as a novel potential agent against echinococcosis in experimentally protoscolex-infected mice. Vet Res.

[9] Luo YP, Zhang Y, Zhang HM, Zhang H, Zhang L, Yu HJ (2018). Anti-parasitic effects of water-soluble alkaloid fractions from ethanolic extracts of Sophora moorcroftiana seeds in Caenorhabditis elegans. Chin J Nat Med.

[10] Li X, Han J, Zhu R, Cui R, Ma XM, Dong K (2016). Life span and motility effects of ethanolic extracts from Sophora moorcroftiana seeds on Caenorhabditis elegans. Pharmacogn Mag.

[11] Ma XM, Yu HJ, Deng Y, Lu YP, Tian WH, An FY (2009). Antitumor effects of ethanolic extracts from Sophora moorcroftiana seeds in mice. Iran Red Crescent Me.

[12] Huang XB, Yuan LW, Shao J, Yang Y, Liu Y, Lu JJ (2021). Cytotoxic effects of flavonoids from root of Sophora flavescens in cancer cells. Nat Prod Res.

[13] Su G, Yang WK, Meng WB, Wu QH, Luo YP, Ma XM (2018). Anti-proliferation effects of ethanolic extracts from Sophora moorcroftiana seeds on human hepatocarcinoma HepG2 cell line. Nat Prod Res.

[14] Ma X, Luo Y, Yu H, Yan C. Ethanolic extracts of Sophora moorcroftiana seeds induce apoptosis of human stomach cancer cell line SGC-7901 in vitro. Afr J Biomed Res 2006. 5(18).

[15] Wang F, Osawa T, Tsuchida R, Yuasa Y, Shibuya M (2011). Downregulation of receptor for activated C-kinase 1 (RACK1) suppresses tumor growth by inhibiting tumor cell proliferation and tumor-associated angiogenesis. Cancer Sci.

[16] Kadzik RS, Homa KE, Kovar DR (2020). F-Actin Cytoskeleton network self-organization through competition and cooperation. Annu Rev Cell Dev Bi.

[17] Pollard TD (2016). Actin and actin-binding proteins. Cold Spring Harb Perspect Biol.

[18] Farniev VM, Shmelev ME, Shved NA, Gulaia VS, Biktimirov AR, Zhizhchenko AY (2022). Nanomechanical and morphological afm mapping of normal tissues and tumors on live brain slices using specially designed embedding matrix and laser-shaped cantilevers. Biomedicines.

[19] Mashima T, Naito M, Noguchi K, Miller DK, Nicholson DW, Tsuruo T (1997). Actin cleavage by CPP-32/apopain during the development of apoptosis. Oncogene.

[20] Kothakota S, Azuma T, Reinhard C, Klippel A, Tang J, Chu K (1997). Caspase-3-generated fragment ofgelsolin: effector of morphological change in apoptosis. Science.

[21] Utsumi T, Sakurai N, Nakano K, Ishisaka R (2003). C-terminal 15 kDa fragment of cytoskeletal actin is posttranslationally N-myristoylated upon caspase-mediated cleavage and targeted to mitochondria. FEBS Lett.

[22] McCleland ML, Adler AS, Shang YL, Hunsaker T, Truong T, Peterson D (2012). An integrated genomic screen identifies LDHB as an essential gene for triple-negative breast cancer. Cancer Res.

[23] Mccleland ML, Adler AS, Deming L, Cosino E, Lee L, Blackwood EM (2013). Lactate dehydrogenase B is required for the growth of KRAS-dependent lung adenocarcinomas. Clin Cancer Res.

[24] Zha X, Wang F, Wang Y, He S, Jing Y, Wu X (2011). Lactate dehydrogenase B is critical for hyperactive mTOR-mediated tumorigenesis. Cancer Res.

[25] Isozaki Y, Hoshino I, Nohata N, Kinoshita T, Akutsu Y, Hanari N (2012). Identification of novel molecular targets regulated by tumor suppressive miR-375 induced by histone acetylation in esophageal squamous cell carcinoma. Int J Oncol.

[26] Ren F, Wu H, Lei Y, Zhang H, Liu R, Zhao Y (2010). Quantitative proteomics identification of phosphoglycerate mutase 1 as a novel therapeutic target in hepatocellular carcinoma. Mol Cancer.

[27] Jiang X, Sun Q, Li H, Li K, Ren X (2014). The role of phosphoglycerate mutase 1 in tumor aerobic glycolysis and its potential therapeutic implications. Int J Cancer.

[28] Sanzey M, Abdul Rahim SA, Oudin A, Dirkse A, Kaoma T, Vallar L (2015). Comprehensive analysis of glycolytic enzymes as therapeutic targets in the treatment of glioblastoma. PLoS ONE.

[29] Bonifati V, Oostra BA, Heutink P (2004). Linking DJ-1 to neurodegeneration offers novel insights for understanding the pathogenesis of Parkinson’s disease. J Mol Medul.

[30] Zhang GQ, He C, Tao L, Liu F (2015). Role of DJ-1 siRNA in reverse sensitivity of breast cancer cells to chemotherapy and its possible mechanism. Int J Clin Exp Pathol.

[31] Zhu H, Liao SD, Shi JJ, Chang LL, Tong YG, Cao J (2014). DJ-1 mediates the resistance of cancer cells to dihydroartemisinin through reactive oxygen species removal. Free Radic Biol Med.

[32] Saeed U, Ray A, Valli RK, Kumar AMR, Ravindranath V (2010). DJ-1 loss by glutaredoxin but not glutathione depletion triggers daxx translocation and cell death. Antioxid Redox Signal.

[33] Im JY, Lee KW, Woo JM, Junn E, Mouradian MM (2012). DJ-1 induces thioredoxin 1 expression through the Nrf2 pathway. Hum Mol Genet.

[34] Hinkle DA, Mullett SJ, Gabris BE, Hamilton RL (2011). DJ-1 expression in glioblastomas shows positive correlation with p53 expression and negative correlation with epidermal growth factor receptor amplification. Neuropathology.

[35] Zhu XL, Sun W, Lei WB, Zhuang HW, Hou WJ, Wen WP (2015). DJ-1-induced phosphatase and tensin homologue downregulation is associated with proliferative and invasive activity of laryngeal cancer cells. Mol Med Rep.

[36] Park SH, Chung YM, Lee YS, Kim HJ, Yoo YDJCCR (2001). Antisense of human peroxiredoxin II enhances radiation-induced cell death. Clin Cancer Res.

[37] Lu W, Fu Z, Wang H, Feng J, Wei J, Guo J (2014). Peroxiredoxin 2 is upregulated in colorectal cancer and contributes to colorectal cancer cells’ survival by protecting cells from oxidative stress. Mol Cell Biochem.

[38] Shiota M, Yokomizo A, Kashiwagi E, Takeuchi A, Fujimoto N, Uchiumi T (2011). Peroxiredoxin 2 in the nucleus and cytoplasm distinctly regulates androgen receptor activity in prostate cancer cells. Free Radic Biol Med.

[39] Lee KW, Lee DJ, Lee JY, Kang DH, Kwon J, Kang SW (2011). Peroxiredoxin II restrains DNA damage-induced death in cancer cells by positively regululating JNK-dependent DNA repair. J Biol Chem.

[40] Hirahashi M, Koga Y, Kumagai R, Aishima S, Taguchi K, Oda Y (2014). Induced nitric oxide synthetase and peroxiredoxin expression in intramucosal poorly differentiated gastric cancer of young patients. Pathol Int.

[41] Furuta J, Nobeyama Y, Umebayashi Y, Otsuka F, Kikuchi K, Ushijima T (2006). Silencing of peroxiredoxin 2 and aberrant methylation of 33 CpG islands in putative promoter regions in human malignant melanomas. Cancer Res.

[42] Liao XH, Li YQ, Wang N, Zheng L, Xing WJ, Zhao DW (2014). Re-expression and epigenetic modification of maspin induced apoptosis in MCF-7 cells mediated by myocardin. Cell Signal.

[43] Wu CT, Wang WC, Chen MF, Su HY, Chen WY, Wu CH (2014). Glucose-regulated protein 78 mediates hormone-independent prostate cancer progression and metastasis through maspin and COX-2 expression. Tumour Biol.

[44] Kim M, Ju H, Lim B, Kang C (2012). Maspin genetically and functionally associates with gastric cancer by regulating cell cycle progression. Carcinogenesis.

[45] Alvarez Secord A, Darcy KM, Hutson A, Huang Z, Lee PS, Jewell EL (2011). The regulation of MASPIN expression in epithelial ovarian cancer: asociation with p53 status, and MASPIN promoter methylation: a gynecologic oncology group study. Gynecol Oncol.

[46] Panou M, Kavantzas N, Sergentanis T, Sakellariou S, Agrogiannis G, Chatzipantelis P (2013). Estimation of maspin’s subcellular localization in invasive ductal breast cancer via light microscopy and computerized image analysis: a comparative study. J BUON.

[47] Machowska M, Wachowicz K, Sopel M, Rzepecki R (2014). Nuclear location of tumor suppressor protein maspin inhibits proliferation of breast cancer cells without affecting proliferation of normal epithelial cells. BMC Cancer.

