# Supplementary material for: Ethanolic extract from Sophora moorcroftiana inhibit cell proliferation and alter the mechanical properties of human cervical cancer
Source: BMC Complement Med Ther. 2024 Jun 3;24:212. doi: 10.1186/s12906-024-04502-5 (PMC11149180; doi:10.1186/s12906-024-04502-5)
Supplement: Supplementary file 1 — Supplementary Material 1 [file 12906_2024_4502_MOESM1_ESM.docx]

**Supplementary Information**

Ethanolic Extract from *Sophora Moorcroftiana* Inhibit Cell Proliferation and Alter the Mechanical Properties of Human Cervical Cancer

Manli Guo ^a☨^, Dingcheng Guo ^b☨^, Lingzi Liao ^b☨^, Xiao Zhang ^b^, Zhilong Wang ^b^, Qiaozhen Zhou ^b^, Ping Chen ^d^, Ruiping Li ^b,c^, Bing Han ^a^, Guangjie Bao ^a§^, Baoping Zhang ^b,c§^

^a^ Key Lab of Oral Diseases of Gansu Province, Northwest Minzu University, Northwest new village No.1, Lanzhou 730030, PR China

^b^ School (Hospital) of Stomatology; Lanzhou University, Donggang West Road 199, Lanzhou 730000, PR China

^c^ Gansu Province Key Lab of Maxillofacial Reconstruction and Intelligent Manufacturing, Donggang West Road 199, Lanzhou 730000, PR China

^d^ Chengdu Stomatological Hospital, NO. 17, South Section of Chunxi Road, Jinjiang District, Chengdu 610020, PR China

^§^Corresponding author

Guangjie Bao, Key Lab of Oral Diseases of Gansu Province, Northwest Minzu University, Northwest new village No.1, Lanzhou 730030, PR China

E-mail: helenbgj@sina.com

Baoping Zhang, School (Hospital) of Stomatology Lanzhou University; Gansu Province Key Lab of Maxillofacial Reconstruction and Intelligent Manufacturing, Donggang West Road 199, Lanzhou 730000, PR China

E-mail: zhangbp@lzu.edu.cn

**^☨^**Manli Guo, Dingcheng Guo and Lingzi Liao contributed equally to this paper.

Table S1 The Sophora Moorcroftiana of bioactive contributing to its anticancer activity

|  | Compound | Cancer |
| --- | --- | --- |
| Matrine alkaloids | Matrine | Colon cancer^[1]^, Liver cancer^[2]^, Lung cancer^[3]^ |
|  | Oxymatrine | Colorectal cancer^[4]^, Renal cell carcinoma^[5]^ |
|  | Sophoridine | Gastric cancer^[6]^, Non-small cell carcinoma^[7]^, Breast cancer^[8]^ |
|  | Sophocarpine | Non-small cell carcinoma^[9]^, Colorectal cancer^[10]^ |
|  | Oxysophocarpine | Colorectal cancer^[11]^, Liver cancer^[12]^ |
| Flavonoids | Genistein | Biliary cancer^[13]^, Ovarian cancer^[14]^ |
|  | Pratensein | Non-small cell carcinoma^[15]^ |
|  | Alpinumisoflavone | Lung cancer^[16]^ |
|  | Diosmetin | Non-small cell carcinoma^[17]^ |

Reference:

[1] Du Q, Lin Y, Ding C, et al. Pharmacological activity of matrine in inhibiting colon cancer cells vm formation, proliferation, and invasion by downregulating claudin-9 mediated emt process and mapk signaling pathway[J]. Drug Design, Development and Therapy, 2023: 2787-2804.

[2] Huang Z, Li H, Li Q, et al. Matrine suppresses liver cancer progression and the Warburg effect by regulating the circROBO1/miR‐130a‐5p/ROBO1 axis[J]. Journal of Biochemical and Molecular Toxicology, 2023, 37(10): e23436.

[3] Zhang H, Chen L, Sun X, et al. Matrine: a promising natural product with various pharmacological activities[J]. Frontiers in pharmacology, 2020, 11: 588.

[4] Pan D, Zhang W, Zhang N, et al. Oxymatrine synergistically enhances doxorubicin anticancer effects in colorectal cancer[J]. Frontiers in Pharmacology, 2021, 12: 673432.

[5] Jin Y, Liu J, Liu Y, et al. Oxymatrine inhibits renal cell carcinoma progression by suppressing β-catenin expression[J]. Frontiers in Pharmacology, 2020, 11: 808.

[6] ur Rashid H, Rasool S, Ali Y, et al. Anti-cancer potential of sophoridine and its derivatives: Recent progress and future perspectives[J]. Bioorganic Chemistry, 2020, 99: 103863.

[7] Zhao B, Hui X, Zeng H, et al. Sophoridine inhibits the tumour growth of non-small lung cancer by inducing macrophages M1 polarisation via MAPK-mediated inflammatory pathway[J]. Frontiers in Oncology, 2021, 11: 634851.

[8] Dai L, Wang L, Tan C, et al. Sophoridine Derivatives Induce Apoptosis and Autophagy to Suppress the Growth of Triple‐Negative Breast Cancer through Inhibition of mTOR Signaling[J]. ChemMedChem, 2022, 17(1): e202100434.

[9] Luo D, Dai X, Tian H, et al. Sophflarine A, a novel matrine-derived alkaloid from Sophora flavescens with therapeutic potential for non-small cell lung cancer through ROS-mediated pyroptosis and autophagy[J]. Phytomedicine, 2023, 116: 154909.

[10] Yang Y, Zhao M, Kuang Q, et al. A Comprehensive Review of Phytochemicals Targeting Macrophages for the Regulation of Colorectal Cancer Progression[J]. Phytomedicine, 2024: 155451.

[11] Li J J, Wang J H, Tian T, et al. The liver microenvironment orchestrates FGL1-mediated immune escape and progression of metastatic colorectal cancer[J]. Nature communications, 2023, 14(1): 6690.

[12] Yang Y, Sun M, Li W, et al. Rebalancing TGF‐β/Smad7 signaling via Compound kushen injection in hepatic stellate cells protects against liver fibrosis and hepatocarcinogenesis[J]. Clinical and Translational Medicine, 2021, 11(7): e410.

[13] Geng Y, Chen S, Yang Y, et al. Long-term exposure to genistein inhibits the proliferation of gallbladder cancer by downregulating the MCM complex[J]. Science Bulletin, 2022, 67(8): 813-824.

[14] Ning Y, Feng W, Cao X, et al. Correction to: Genistein inhibits stemness of SKOV3 cells induced by macrophages co-cultured with ovarian cancer stem-like cells through IL-8/STAT3 axis[J]. Journal of Experimental & Clinical Cancer Research, 2021, 40: 1-2.

[15] Zhou Y, Wu C, Qian X, et al. Multitarget and multipathway regulation of zhenqi fuzheng granule against non-small cell lung cancer based on network pharmacology and molecular docking[J]. Evidence-Based Complementary and Alternative Medicine, 2022, 2022.

[16] Namkoong S, Kim T J, Jang I S, et al. Alpinumisoflavone induces apoptosis and suppresses extracellular signal-regulated kinases/mitogen activated protein kinase and nuclear factor-κB pathways in lung tumor cells[J]. Biological and Pharmaceutical Bulletin, 2011, 34(2): 203-208.

[17] Chen X, Wu Q, Chen Y, et al. Diosmetin induces apoptosis and enhances the chemotherapeutic efficacy of paclitaxel in non‐small cell lung cancer cells via Nrf2 inhibition[J]. British journal of pharmacology, 2019, 176(12): 2079.


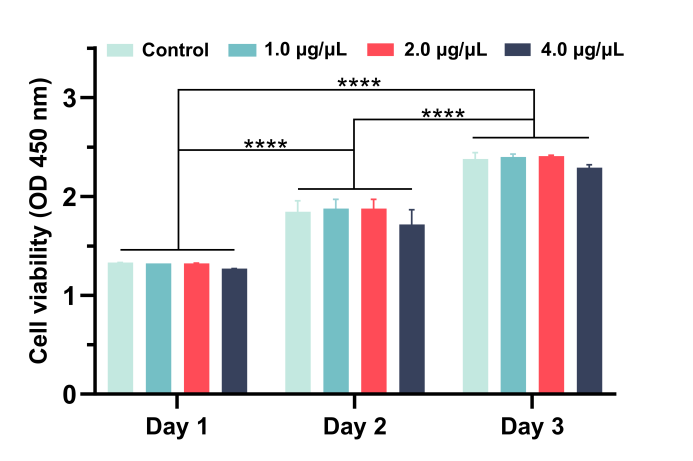


Figure S1 The effect of ethanolic extract from Sophora Moorcroftiana Seeds on the activity of mouse fibroblasts L929 cells line
